# *Marham‐Mafasel* decrease joint inflammation and *IL‐1β* gene expression in rheumatoid arthritis animal model

**DOI:** 10.1002/vms3.430

**Published:** 2021-05-03

**Authors:** Mohammad Majidi, Fatemeh Heidarnejad, Mohsen Naseri, Shahin Bonakdar, Maryam Salimi, Roya Yaraee

**Affiliations:** ^1^ Department of Tissue Engineering & Regenerative Medicine Faculty of Advanced Technologies in Medicine Iran University of Medical Sciences Tehran Iran; ^2^ Department of Immunology Medical Faculty Shahed University Tehran Iran; ^3^ National Cell Bank Department Pasteur Institute of Iran Tehran Iran; ^4^ Traditional Medicine Clinical Trial Research Center Shahed University Tehran Iran; ^5^ Department of Biology and Anatomical Sciences Faculty of Medicine Shahid Beheshti University of Medical Sciences Tehran Iran; ^6^ Immunoregulation Research Center Shahed University Tehran Iran

**Keywords:** adjuvant, cytokine, herbal medicines, joint inflammation

## Abstract

**Background:**

Rheumatoid arthritis (RA) is a systemic chronic disease with synovial membrane, tendon and articular tissue inflammation. Current treatments of RA have many side effects and are quite expensive. Today, new treatments procedures and inexpensive herbal drugs are developed. *Marham‐Mafasel* is mainly made out of two traditional herbs (*Arnebia euchroma* and *Martricaria chamomilla*).

**Objective:**

In this study, for the first time, the impact of *Marham‐Mafasel* on joint inflammation, histopathological changes and *IL‐1β* gene expression was evaluated in RA animal model.

**Methods:**

The RA was induced by a single s.c. injection of 0.1 ml Freund's complete adjuvant into the left hind footpad. In continuous, 15 RA male Wistar rats were used in three groups: I: Control; II: Treatment I (Piroxicam) and III: Treatment II (*Marham‐Mafasel*). The volume of the hind paw was measured every day from 0 to 19 using water changed volume approach. The inflammation in the joint was evaluated using histopathology assay and gene expression of *IL‐1β* was evaluated with use of Real‐Time PCR.

**Results:**

Hind paw swelling of *Marham‐Mafasel* at days 10th and 19th was reduced compared with the control group (*p* < 0.05). There was no statistically difference in histological degrading and changes index in three groups (*p* ≥ 0.05). Relative expression of *IL‐1β* in *Marham‐Mafasel* group was significantly decreased compared with other groups.

**Conclusion:**

The co‐administration of *M. Chamomile* and *A. euchro*ma, called *Marham‐Mafasel*, decreases *IL‐1β* gene expression that leads to a reduction in inflammation in rheumatoid arthritis (RA) animal model.

## Graphical abstract

Rheumatoid arthritis (RA) is a systemic chronic disease with synovial membrane, tendon and articular tissue inflammation. The co‐administration of M. Chamomile and A. euchroma, called *Marham‐Mafasel*, decrease IL‐1β gene expression that leads to a reduction in inflammation in rheumatoid arthritis (RA) animal model.

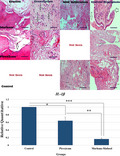

## 1 INTRODUCTION

Rheumatoid arthritis (RA) is an autoimmune disease characterized by systemic complications, progressive disability, pain, progressive course affecting articular, mortality and socioeconomic costs (Combe, 2009; El Miedany et al., 2008). RA is the consequence of a disorder of immune system function and its reason is still controversial (Entezami et al., 2011). RA is found to affect up to 1% of individuals in developed countries (Suresh & Shetty, 2018). The most prevalent involved joints are feet and hands such as knees, ankles, shoulders, elbows and wrists. As a type of chronic autoimmune disease, RA causes erosive joint damage, functional impairment, progressive bone and cartilage destruction in the vast majority of the patients (Combe, 2009; El Miedany et al., 2008; Venables & Maini, 2014). Antibodies are found as an important driver in the pathogenesis of RA. Anti‐citrullinated protein antibodies (ACPAs) and rheumatoid factor (RF) are proved useful for RA diagnosis and reported to be present in nearly 70% of the patients (Ursum et al., 2009). The *IL‐1β* is an inflammatory cytokine that has association with elevated level of RA (Firestein & Malnnes, 2017). Proteolytic degradation of joint cartilage and bone is generally irreversible and a characteristic feature of joint destruction. In RA, inflammatory cells infiltrate the synovial membrane and induce bone and cartilage degradation. Histologic observation has shown the stage of RA lesion and the therapeutic effects of the drugs (Fassbender, 1983; Shegarfi et al., 2012). RA is characterized by synovial inflammation in which Fibroblast‐like synoviocytes (FLS) in synovial joint play a central role in the production of pro‐inflammatory cytokines and chemokine (Bartok & Firestein, 2010; Bottini & Firestein, 2013). The course of the disease is varied according to the presence or absence of autoantibodies, frequency of swollen joints, the severity of inflammatory process and genetic background (Gossec et al., 2010; Heidari, 2011). Therapeutics available against RA can help symptomatic relief (no steroidal anti‐inflammatory drugs; NSAIDs) or modifies the disease progression (disease‐modifying anti‐rheumatic drugs; DMARDs). The prolonged use of drugs left the patients susceptible to gastrointestinal, anaemia, renal impairment and cardiovascular diseases (Guo et al., 2018; Wong, 2019). Today, the natural agents with therapeutic potential have increased attention and tested for the treatment of arthritis (Dudics et al., 2018; Zhang & Dai, 2012). Interestingly, about 59% of Pharmacopoeia is obtained from herbal plants (Swerdlow, 2000). In the history, Persian Medicine (PM) dates back to 10,000 years ago which was one of the burgeoning schools of medicine (Ghaffari et al., 2014, 2017). PM with several thousands of manuscripts, famous scientists and verbal sources in different languages can be helpful in the treatment of various diseases and in the development of new drugs by using modern technology (Ahmadi et al., 2009; Goshtasebi et al., 2015; Pasalar et al., 2013).

Some Traditional Iranian Medicines like *Marham‐Mafasel* have accepted to be effective against RA. *Marham‐Mafasel* is a mixture of two herbs, namely, *Arnebia euchroma*(*A. euchroma*) and *Matricaria chamomilla* (*M. chamomilla*) (Soltanian et al., 2010). *A. euchroma* is a traditional herbal medicine that has some functions, including immune‐modulatory, antifungal and anti‐inflammatory (Siavash et al., 2016). *M*. *chamomilla* was previously administered in the USA, Europe, Western Asia, Australia and other countries (Mehmood et al., 2015). Also, previous studies on herbal medicines have revealed a significant decrease in the inflammation of oral mucositis and atopic eczema in the rat animal model (Ferreira et al., 2015; Srivastava et al., 2010; Tavakoli Ardakani et al., 2016). So, these herbal medicines have the potential to reduce the inflammation and inflammatory factors in RA patients. However, there is no study on anti‐inflammatory activities of co‐administration of these herbs on RA animal model. Therefore, in the present study, the effects of *Marham‐Mafasel* (an herbal ointment) on the reduction in joint inflammation, histopathological changes and *IL‐1β* gene expression in the hind paw of adjuvant‐induced RA rat model was evaluated.

## 2 MATERIALS

### 2.1 Animals and RA induction

The study procedures were confirmed by the Research Ethics Committee of Shahed University of Medical Scinedces, Tehran Iran (IR.Shahed.REC.1396.104). Fifteen Male Wistar rat weighing 250–300 g were obtained from Pasteur Institute of Iran and kept for 7 days in the cages prior to assays. The rats were maintained in the standard laboratory condition with free access to food and water ad libitum. The RA was induced by a single s.c. injection of 0.1 ml Freund's complete adjuvant into the left hind footpad (Ahmed et al., 2018). Alterations in paw swelling are a common indicator that used to identify the anti‐arthritis effects of drugs in the Rat Adjuvant‐Induced Arthritis (AIA) model. The rats were divided into three groups: I: Control; II: Treatment I (Piroxicam) and III: Treatment II (*Marham‐Mafasel*). All animals received the herbal ointment and Piroxicam (0.5%) directly in affected site every day in which administration of drug was started from day 0 to day 19 of RA disease.

### 2.2 Drug preparation

*Marham‐Mafasel* is a mixture of two herb extracts, namely, *A*. *euchroma* and *M. chamomilla* (Soltanian et al., 2010), and purchased from special drugstore. Piroxicam was purchased from Razak laboratory of Iran.

### 2.3 Evaluation of hind paws oedema and body weight

The volume of the hind paw was measured on days 0, 2, 4, 6, 8, 10, 12, 14, 16, 18 and 19. The change in a water volume of the graduated cylinder was used for the evaluation of paws oedema (Fereidoni et al., 2000). The paw swelling indexes (%) was calculated by subtraction of each day volume from day 0 and divides the equal from each day (Gupta et al., 2014).

### 2.4 Radiography

On the day 19, all the rats were sacrificed. Lateral and anterior radiographic images from left hind paws were taken to evaluate the cartilage and bone damages with a 55 kVp exposure for 6.4 mAs (Siemens Multiphos 15, Mumbai, and Maharashtra, India).

### 2.5 RNA Isolation and cDNA synthesis

The one sample of cartilage and bone tissue of talus from each group were dissected and kept at –80°C until the RNA isolation. The total RNA was obtained with use of RNAqueous®‐Micro Kit according to the manufacturer's instruction (Ambion). The genomic DNA was removed with use of DNase (Qiagen) at 37°C for 15 min. The RNA concentration was quantified by the A260/A280 ratio with use of a Nano Drop ND‐2000. All the extracted RNAs, from three groups, were stored at −80°C. cDNA was synthesized with the use of PrimeScript RT reagent Kit (TaKaRa). Briefly, a mixture of total RNA (2.5 ng), oligo (dT), RT enzyme, random hexamer and buffer was heated at 37°C for 15 min. The cDNAs were stored at −20°C until Real‐Time PCR was perform.

### 2.6 Real‐time PCR

The **g**ene expression was evaluated with the use of SYBR Green PCR Kit (TaKaRa) and a Step One instrument (Applied Biosystem). All reactions were carried out in duplicate and in a total volume of 20 μl for 50 cycles. In this study, quantitative real‐time PCR was used to assess the expression of the *IL‐1β* gene (Forward: 5' ‐ TCATTGTGGCTGTGGAGAAG ‐ 3’ Reverse: 5' ‐ CACTAGCAGGTCGTCATCATC ‐ 3'). *IL‐1β* was an inflammatory cytokine that was used for evaluation of drug effects and beta‐actin gene (Forward: 5' ‐ GTCCACCTTCCAGCAGATG ‐ 3’ Reverse: 5' ‐ GCTCAGTAACAGTCCGCCTAG ‐ 3') used as endogenous control. Three different software were used for primer design including Beacon Designer, Gene Runner and Primer Express. The condition of each cycle was as follows: 95°C for 30 s (holding time), 95°C for 5 s (denaturing) and 60°C for 30 s (annealing and extension). The melting curve analysis was performed by 95°C in the first step then cooled to 60°C and stepwise heated to 95°C with a ramp rate of 0.3°C. Finally, the relative gene expression of *IL‐1β* was calculated with use of ∆∆Ct method.

### 2.7 Histopathology

On the day 19, all the rats were sacrificed, tibio‐femoral joints were removed, fixed immediately in 10% neutral buffered formalin and decalcified in 10% formic acid. The samples were then dehydrated, embedded in paraffin wax and serially sectioned at a thickness of 5 μm. The sections were stained with Hematoxylin and Eosin to evaluate for structural changes and cellular infiltration. Histological changes and degrading index of sections were scored as described previously (Srivastava et al., 2010).

### 2.8 Statistical analysis

Histological and degrading Index was analysed using Chi‐squared test. The relative levels of mRNA derived in different groups were analysed by REST 2009 Software (Qiagen, Hilden, Germany). The normality of hind paw swelling was analysed by Kolmogorov–Smirnov (KS) test and their significance was analysed by one‐way ANOVA (Tukey's post‐hoc test) and expressed as mean ± *SD*. The analyses were performed with use of the SPSS Statistical program, version 22 (SPSS). The comparison tests were considered to be significantly different at *p* < 0.05.

## 3 RESULTS

### 3.1 Measurement of arthritis swelling

**FIGURE 1:**
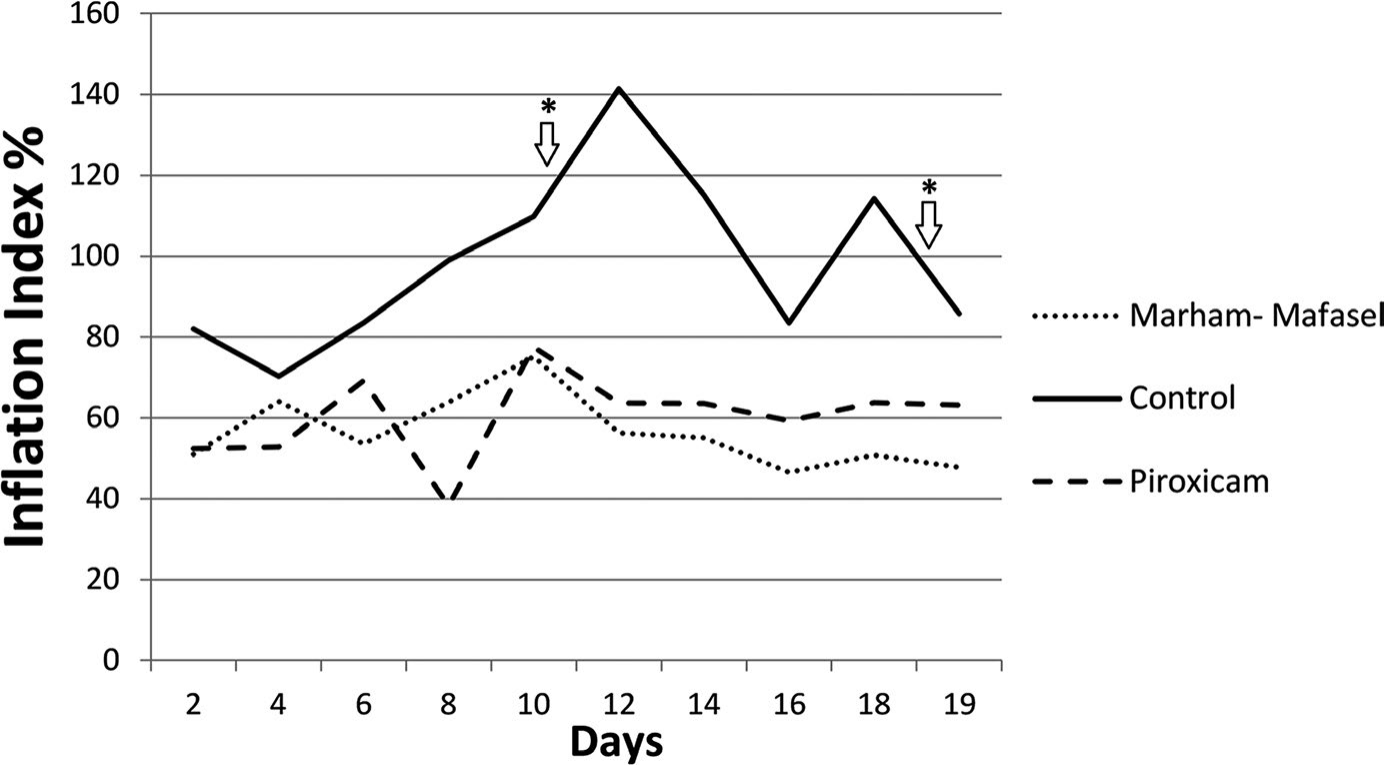
The mean of hind paw swelling index has been shown for the three groups of control, Piroxicam and *Marham‐Mafasel*. Hind paw swelling of *Marham‐Mafasel* at day 10th and 19th was reduced compared with the control group (*p* < 0.05). *The significant days between the groups

As shown in Figure 1, the hind paw swelling was showed in three groups from days 0 to 19 (Figure 1). The most important days in the animal model of RA are day 10th: acute inflammation and day19th: chronic inflammation. The lowest rate of oedema in the control, Piroxicam and *Marham‐Mafasel* group was on days 4, 8 and 19, respectively, which are different with each other. Hind paw swelling of *Marham‐Mafasel* at day 10th and 19th was reduced compared with the control group (*p* < 0.05). Also, the mean of the inflammation index in the Piroxicam group was more than *Marham‐Mafasel,* but the difference was not statistically significant (*p* ≥ 0.05) (Figure 1).

### 3.2 Radiography

**FIGURE 2:**
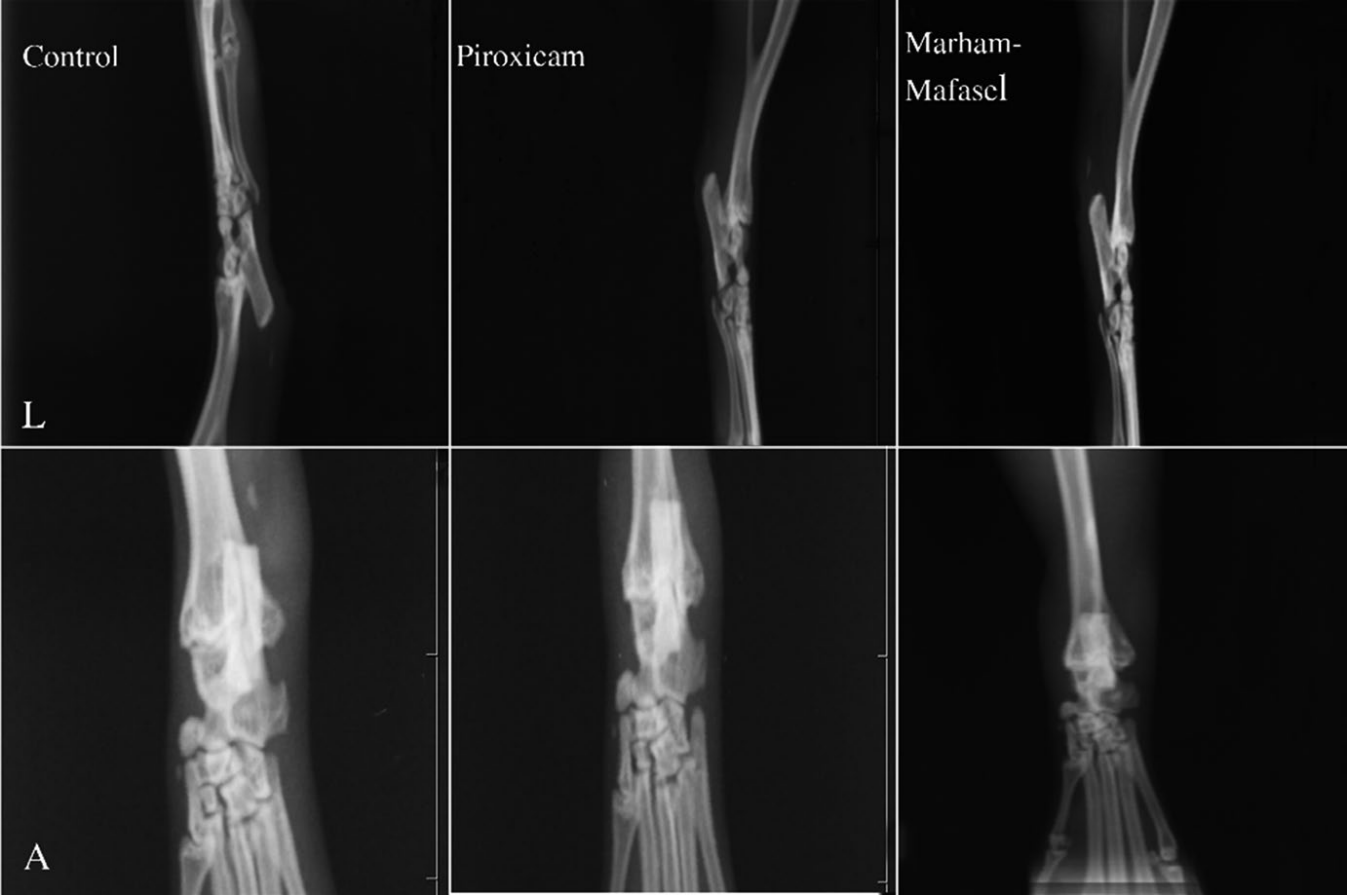
On the day 19, all the rats were sacrificed. Radiography Images were taken from lateral and anterior aspect of left hind paws. There was no statistically significant difference between the three groups – control, Piroxicam and *Marham‐Mafasel*. (L; Lateral view, A; Anterior view)

On the 19‐day of experiment, all the rats were sacrificed then immediately lateral and anterior radiographic images were taken from left hind paw. The radiological changes in the bone and cartilage were evaluated in which there was no statistically difference between the groups (*p* ≥ 0.05) (Figure 2).

### 3.3 Histopathology assay

#### 3.3.1 Histological index

**FIGURE 3:**
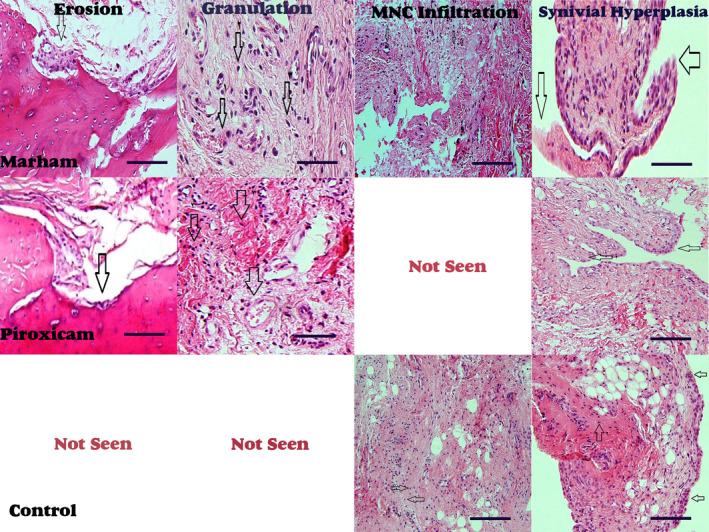
Histopathological degrading index including thinning of the cartilage layer, low erosion, extreme erosion and breaking of cartilage was analysed in three groups of control, Piroxicam and *Marham‐Mafasel*. There was no statistically significant difference in histological changes in all three groups (*p* > 0.05) (Table. 2 and Figure 3). Not seen; not observed in this group

In this study, the grading of histological index, including synovial hyperplasia, pannus formation, mild cell migration, mononuclear cell infiltration and granulation tissue, in all groups was analysed. Our results demonstrated that pannus formation and granulation tissue occurred in Piroxicam and *Marham‐Mafasel* groups but not observed in the control group. Mild cell migration index occurred in all of the samples in Piroxicam groups compared with other groups. Our results showed that mononuclear cell infiltration occurred identically in all groups (*p* > 0.05). Taken together, there was no significantly difference in histological changes in all three groups (*p* > 0.05) (Table. 1 and Figure 3).

**TABLE 1:** The grading of histological index, including synovial hyperplasia, pannus formation, mild cell migration, mononuclear cell infiltration and granulation tissue, was performed in all three groups

	Synovial hyperplasian/5	Pannus formation/5	Mild Cell MigrationN/5	Mononuclear Cell infiltration *N*/5	Granulation tissue *N*/5
Control	3	0	3	5	0
Piroxicam	4	1	5	5	1
*Marham‐Mafasel*	3	1	4	5	1

Note

There was no significant difference in histological changes between groups (*p* > 0.05). *N*; the numbers of Wistar rat in each groups.

#### 3.3.2 Histological degrading index

In this study, the histological degrading index including thinning of the cartilage layer, low erosion, extreme erosion and breaking of cartilage in all groups was analysed. Our results showed that the thinning of the cartilage layer, low and extreme erosion were not observed in *Marham‐Mafasel* samples but observed in Piroxicam group. The extreme erosion was not observed in all samples of three groups. There was no statistically significant difference in histological changes in all three groups (*p* > 0.05) (Table. 2 and Figure 3).

**TABLE 2:** The histological degrading index including thinning of the cartilage layer, low erosion, extreme erosion and breaking of cartilage was performed in all three groups

	Thinning of the Cartilage layer *N*/5	Low erosion *N*/5	Extreme erosion *N*/5	Breaking of cartilage *N*/5
Control	0	0	0	0
Piroxicam	1	1	0	1
*Marham‐Mafasel*	0	0	0	1

Note

There was no statistically significant difference in histological changes between groups (*p* > 0.05). *N*; the numbers of Wistar rat in each groups.

### 3.4 *IL‐1β* gene expression

**FIGURE 4:**
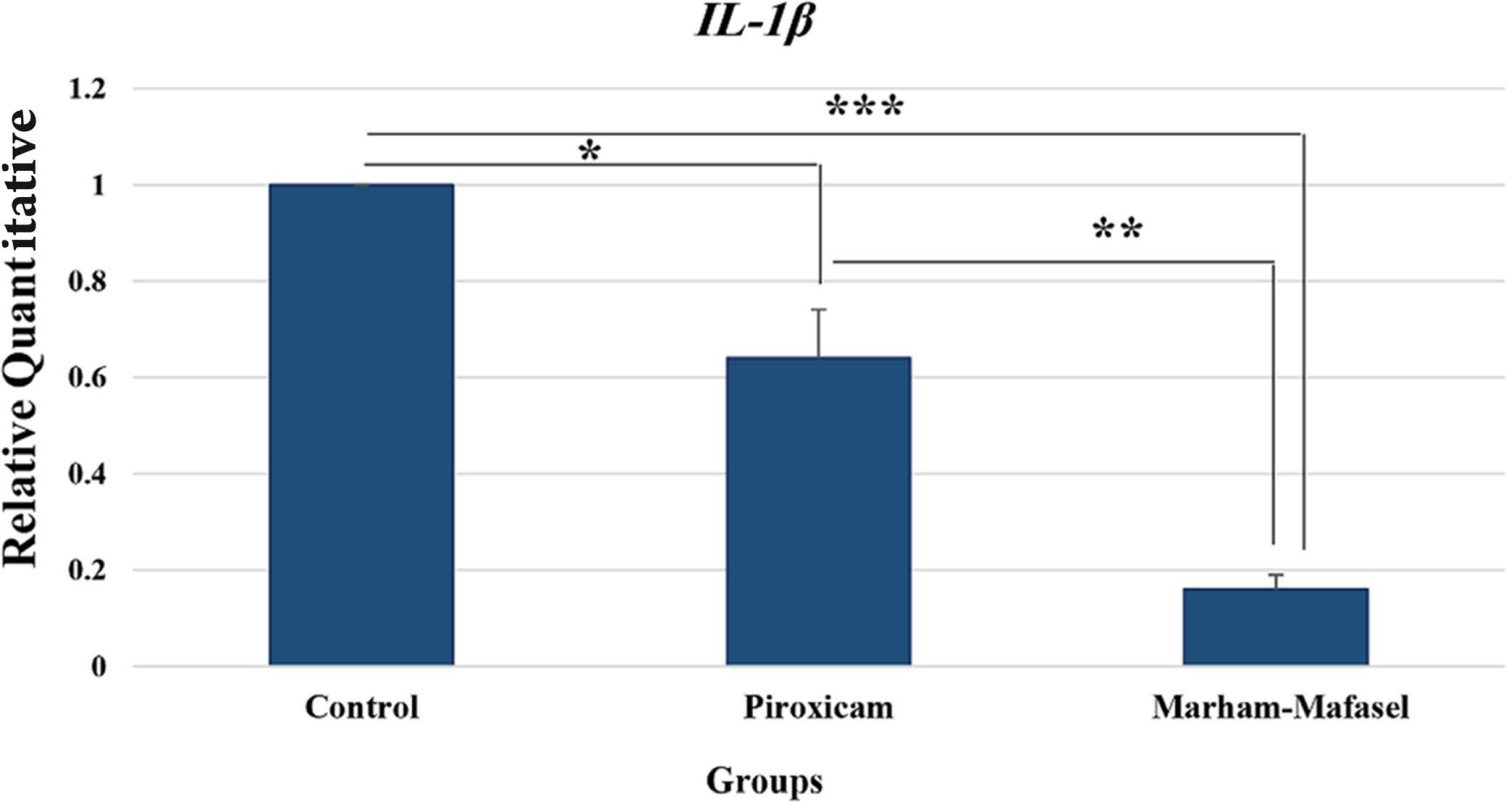
The relative quantitative gene expression of *IL‐1β* (Mean ± *SEM*) was calculated with the use of ΔΔCT method. The relative expression of *IL‐1β* in *Marham‐Mafasel* was decreased compared with Piroxicam and in Piroxicam was decreased compared with control (*p* < 0.05); *p < 0.05, **p < 0.01, **p < 0.001

This study revealed that the relative gene expression of *IL‐1β* in *Marham‐Mafasel* was decreased compared with Piroxicam and in Piroxicam was decreased compared with control (*p* < 0.05) (Figure 4).

## 4 DISCUSSION

RA is a chronic systemic disease which can cause multi‐joints damages, chronic joint inflammation, pain, joint deformity and disability (Kim et al., 2017). Rat Adjuvant‐Induced Arthritis (AIA) model is a reliable, reproducible and easy animal model of arthritis with a limited duration. AIA has been widely used to study the anti‑inflammatory activity of drugs because of the similarity of pathology with human RA (Bolon et al., 2010; Kannan et al., 2005). AIA is a classic experimental model of RA and shares many pathologic features with RA such as synovial hyperplasia, cartilage damage and excessive inflammation (Cai et al., 2017). The synovial fibroblasts are directly involved in cartilage and bone destruction by a regulatory network of cytokines that implicate inflammation (Choy, 2012; Guo et al., 2018). The common treatments of RA are typical with immunosuppressant that often cause adverse impacts, whereas herbal products are considered safe and effective (Swerdlow, 2000). These drugs prevent arthritic joints from the tissue damage, bone breakdown and also be highly tolerated and convenient for the patients (Nanjundaiah et al., 2013). The results of hind paw oedema assay in this study suggested that the *Marham‐Mafasel* treatment has anti‐oedema activities. The effect of *Marham‐Mafasel* on the reduction in pain on primary knee osteoarthritis has been identified (Soltanian et al., 2010). Our study was in one direction with Soltanian et al. (2010 **)** that revealed the anti‑inflammatory activity and benefits of mixture solution of *A. euchroma* and *M*. *chamomilla* on pain relief, physical function and stiffness on chronic osteoarthritis of the knee (Soltanian et al., 2010); however, in present study, we evaluated the other aspect of molecular and pathological biology. Also, this result was consistent with Amraei et al. (2015 **)** that showed *M. chamomilla* has more anti‐inflammatory activity than traditional anti‐inflammatory drugs including indomethacin and dexamethasone (Amraei et al., 2015). Also, Fan et al., 2012 showed that *A*. *euchroma* has anti‐arthritic function by suppressing inflammation and paw swelling development in experimental animal model (Fan et al., 2012). Fan observed that 2.5 mg/kg of *A. euchroma* can significantly reduce the paw swelling at the late period of the experimental arthritis (from days 13 to 29) (Fan et al., 2012). The results of Fan were nearly in agreement with our data that showed paw swelling was decreased at the last period of the arthritis (from days 10 to 19), But in our study, due to the co‐administered of *A. euchroma* and *M. chamomilla*, we had faster reduction in the paw swelling at starting point and also the duration of the disease.

The main pathological characteristics of the RA are synovial cell hyperplasia, infiltration of inflammatory cells, cartilage degeneration, bone erosion and pannus formation that also occurred in AIA rats (Amraei et al., 2015). The histopathological index in the articular structure was confirmed by H&E staining (Taranov et al., 2016). In the present study, no obvious differences were noted among the *Marham‐Mafasel*, Piroxicam and control groups. On this regard, Fan et al revealed that there was no improvement of cartilage and bone destruction in a low dose of the *A. euchroma* but the histopathological index was reduced after receiving a high dose (Fan et al., 2012). It should also be noted that there are no histopathological index reports that show the impact of co‐administration of *M. chamomilla*, *M.chamomilla* and *A. euchroma* on treatment of arthritis. We considered this result to the fact that we study an AIA model in which little histological injury had yet occurred. Our purpose was to evaluate the joint preservation effect in early stage of the disease and also determine the severity of the inflammation. Therefore, for future studies, we suggest the evaluation of different concentrations of the co‐administered of these herbs on histopathological changes in AIA rat models.

The pathophysiology of the RA is not completely understood yet (Imboden, 2009), But previous studies have confirmed that pro‐inflammatory cytokines, such as *TNF‐α*, *IL‐1* and *IL‐6,* mainly produced by increased numbers of macrophages, are responsible for the inflammation and joint destruction (Guo et al., 2018). Disease‐modifying anti‐rheumatic drugs (DMARDs), NSAIDs and corticosteroids are commonly used in the treatment of the arthritis patients (Kemper et al., 2011). These drugs are first‐line drug therapies which have an anti‐inflammatory activity against the mentioned cytokines (Kemper et al., 2011). *IL‐1β* is the initiating factor in inflammation to regulate a variety of cytokines, cell adhesion molecules and inflammatory mediators. Previous studies showed that high level of *IL‐1β* was associated with bone erosion and cartilage destruction in RA and manifested additional characteristics of osteoarthritis (OA) which included increased osteophyte complex form (Guo et al., 2018; Wojdasiewicz et al., 2014). Also, previous studies have demonstrated that the *IL‐1* has main role in the pathophysiology of the RA. Elevated concentrations of *IL‐1* in plasma and synovial fluids of the RA patients were detected compared with OA or non‐inflammatory joint disease (Kay & Calabrese, 2004). Given all these facts, the gene expression of *IL‐1* was measured in this study to evaluate the impact of *Marham‐Mafasel* on the severity of the RA. Five biological replications were performed in each group and one sample of each biological replication is selected for testing. *IL‐1β* is an inflammatory cytokine that affected by many factors which may confuse the results then for preventing, we selected one rat of each group and evaluated the *IL‐1β* in duplicate. Our data revealed that the expression of IL‐1β in Marham‐Mafasel group was decreased compared with the Piroxicam and control groups. In agreement with our results, Guimarães et al., 2016 has showed that *M. Chamomile* prevent the inflammation of alveolar bone by reducing the gene expression of *IL‐1β* (Guimarães et al., 2016). Also, Fan et al., 2012 found that *A. euchroma* administered in experimental arthritis has anti‐arthritic activity by reducing the gene expression of *TNF‐α* and *IL‐1β*. It should be noted that there is no study that shows the impact of co‐administration of these compounds on the inflammatory factors (Fan et al., 2012). According to the results of these studies, it can be concluded that *Marham‐Mafasel* with compounds of *M. chamomile* and *A. euchroma* reduces inflammatory reactions and swelling by *IL‐1β* decline.

## 5 CONCLUSIONS

This study, for the first time, has observed that the co‐administration of *M. Chamomile* and *A. euchro*ma, called *Marham‐Mafasel*, decrease the gene expression of *IL‐1β* that leads to a reduction in the inflammation in (AIA) model.

## CONFLICT OF INTEREST

The authors declare no conflict of interest.

## AUTHOR CONTRIBUTION

**Mohammad Majidi:** Conceptualization; Investigation; Methodology; Writing‐original draft. **Fatemeh Heidarnejad:** Data curation; Software; Visualization. **Mohsen Naseri:** Formal analysis; Methodology; Project administration. **Shahin Bonakdar:** Funding acquisition; Methodology; Project administration; Validation. **Roya Yaraee:** Conceptualization; Supervision; Writing‐review & editing.

## AUTHORS CONTRIBUTIONS

M.M., R.Y; Participated in designed experiments and critical revision of the manuscript. M.N., SH.B. F.H., M.S; Performed data collection and analysed the data. All authors revised the manuscript and approved the final manuscript.

## ETHICAL STATEMENT

The experimental animal's process was performed based on guidelines by the Research Ethics Committee of Shahed University of Medical Sciences and Pasteur Institute of Iran, Tehran, Iran.

### Peer Review

The peer review history for this article is available at https://publons.com/publon/10.1002/vms3.430.
